# Fe(III)-Rhamnoxylan—A Novel High Spin Fe(III) Octahedral Complex Having Versatile Physical and Biological Properties

**DOI:** 10.3390/polym14204290

**Published:** 2022-10-12

**Authors:** Anum Hayat, Mohammad Saeed Iqbal, Naveed Ahmad, Nabil K. Alruwaili, Atta ur Rehman

**Affiliations:** 1 Department of Chemistry, Forman Christian College, Lahore 54600, Pakistan; 2 Department of Chemistry, Kinnaird College for women, Lahore 54000, Pakistan; 3 Department of Pharmaceutics, College of Pharmacy, Jouf University, Sakaka 72388, Al-Jouf, Saudi Arabia; 4 Department of Pharmacy, Forman Christian College, Lahore 54600, Pakistan

**Keywords:** Iron(III)-hemicellulose complex, rhamnoxylan, iron supplement, magnetic polymer, hematinic

## Abstract

An iron (III) complex with rhamnoxylan, a hemicellulose from *Salvia plebeia* seeds, was synthesized and characterized by elemental analysis, spectroscopic and magnetic susceptibility measurements, thermal analysis and scanning electron microscopy. The rhamnoxylan was found to be a branched hemicellulose consisting of β-1,4-linked xylose main chain and rhamnose attached to the chain at β-1,3 positions. The complex was found to contain 18.8% *w/w* iron. A high-spin octahedral geometry of Fe^3+^ was indicated by the electronic absorption spectrum of the complex. In other experiments, the complex exhibited good electrical and magnetic properties. In vivo efficacy, as hematinic, of the complex in induced anemia was demonstrated equivalent to that of iron protein succinylate (taken as standard) as evidenced by raised red blood cell count, hemoglobin, hematocrit and total iron in rabbit. The complex was found to be non-toxic with LD50 > 5000 mg kg^−1^ body weight in rabbit. Thus, iron(III)-rhamnoxylan hold the potential for application as hematinic for treatment of iron deficiency anemia.

## 1. Introduction

The iron deficiency anemia is of concern in developed countries and a serious problem in underdeveloped countries. It is caused by low iron diet in developed countries and scarcity of nutritional diet in underdeveloped countries [1]. Iron deficiency causes a drop in hemoglobin level in blood. Typical hemoglobin levels in humans are >13 g dL^−1^ (males) and >12 g dL^−1^ (females); these levels may drop to 3 g dL^−1^ or lower in case of extensive blood loss due to hemorrhage, menstrual flow and bleeding ulcer [2]. In order to treat such conditions iron compounds are administered as therapeutics or included in dietary supplements. The iron salts currently used as therapeutics include ferrous sulphate, ferric chloride, ferric ammonium citrate, ferrous fumarate and complex compounds like ferrous gluconate, iron dextran, iron sorbitex, ferric carboxymaltose, ferric hydroxide polymaltose complex and iron protein succinylate (IPS) [3]. Among these, polymeric iron complexes are considered more useful as simple iron salts, when administered orally, interact with food and other medications resulting in reduced bioavailability [4]. On the other hand, the polymeric complexes are relatively stable and release iron in a sustained manner through ligand exchange processes in the body. Therefore, the complexes are considered beneficial for oral administration. Large molecule polysaccharide metal complexes exhibit higher safety as compared with traditional small molecule metal complexes. Therefore, polysaccharide metal complexes from single polysaccharides can be used as promising novel drugs.

Fe^3+^ forms both low-spin and high-spin octahedral complexes with different ligands. High-spin complexes are formed with weak-field ligands such Cl^−^ or OH^−^ ions. Low-spin and high-spin complexes play important role in spin-cross-over reactions in the biological system. Normally, the low-spin state is more favorable for Fe^3+^ complexes. The proteins having octahedral Fe^3+^ environment with all the six sites occupied include cytochromes and ferredoxins, which are involved in electron transfer processes. The title complex being reported here would mimic the role of these proteins. To the best of our knowledge no high-spin octahedral complex with a hemicellulosic ligand has been reported.

The problem with the currently available iron pharmaceuticals and nutritional supplements is that they cause gastric irritation and low efficacy. Ferric hydroxide polymaltose complex and IPS, considered to be safe [5], also exhibit side effects to varying degrees. Treatment with ferric hydroxide polymaltose is usually associated with diarrhea and lower onset effect [4,5], whereas the IPS may not be tolerated by the patients showing casein allergy. So, it is desirable to design and synthesize new iron complexes having lower toxicity, better tolerance and bioavailability. It has been reported that hemicelluloses have the potential to be used as drug carriers because of being biocompatible, biodegradable and digestible by human [6,7,8]. The present study was designed to investigate the potential of hemicelluloses to bind enough quantity of iron for use as hematinic. For this purpose, rhamnoxylan, a hemicellulosic material isolated from *Salvia plebeian* seeds, commonly known as sage and regionally recognized as ‘Samundarsok’ and ‘Kamarkas’, was used as a model [9].

*S. plebeia* is a herb found along sides the streams and rivers in different parts of the world. The plant’s classification is: Plantae (kingdom), Magnoliophyta (division), Magnoliopsida (class), Lamiales (order), Lamiaceae (family), Salvia (genus) and plebeia (species). The extracts of seeds and leaves of this plant have significant therapeutic value; the popular uses include sore throat, headache, asthma and inflammatory diseases [10]. When seeds of *S. plebeia* are soaked in water, they produce a mucilage mainly consisting of hemicelluloses identified as rhamnoxylans having weighted-average molar mass (Mw) of 2.47 × 10^6^ Daltons containing Xylp 99.32% and Rhap 0.68%. In the present work, it has been hypothesized that rhamnoxylan forms an iron complex having better efficacy, bioavailability and toxicity profile for treating iron deficiency.

## 2. Results and Discussion

### 2.1. Synthesis of Fe(III)-Rhamnoxylan

A dark brown complex was obtained by the experimental procedure as discussed in the methods section. The role of NaOH is to facilitate dissolution of rhamnoxylan during complex formation. In order to remove Fe(OH)_3_, that may have formed due to a reaction of FeCl_3_ with NaOH, the complex was thoroughly washed with dilute HCl. Further washings with methanol and ether afforded a significantly dry product, which was further dried at 60 °C in an oven to a constant weight and ground to obtain a free-flowing powder in good yield (~90%). The complex was sparingly soluble (approx. 1 g in 100 mL) in dimethyl sulfoxide (DMSO) and dimethylformamide (DMF), and insoluble in other organic solvents and water.

### 2.2. Characterization

#### 2.2.1. Elemental Analysis

Elemental analysis of the rhamnoxylan sample and the synthesized Fe(III)-rhamnoxylan complex (Table 1) shows the presence of C and H. The percentages in the complex of these elements (C: 31.1%; H: 5.15%) were lower than those of rhamnoxylan (C: 46.73%; H: 6.45%) as expected due to the presence of iron (18.40%) in the complex. However, the C:H molar ratio was similar in the two materials, which confirmed that iron is bonded to the rhamnoxylan. The AgNO_3_ gave negative test for Cl^−^ ions. The water content in the complex (11.0%) was higher than that in the rhamnoxylan used (5.98%) indicating the hygroscopic nature of the complex.

#### 2.2.2. Monosaccharide Analysis

The monosaccharide analysis in Table 1 clearly indicates the presence of Xyl*p* and Rha*p* in the complex; their percentages are lower than those in pure rhamnoxylan due to the presence of iron as part of the complex. However, the Xyl*p*:Rha*p* ratio in the complex is similar to that in the rhamnoxylan. This effect is also noticeable in the relatively higher molar mass of the complex (2.52 × 10^6^ D) compared to that of the rhamnoxylan (2.47 × 10^6^ D) as determined by GPC (Table 1).

#### 2.2.3. FT-IR and Raman Analysis 

**Figure 1:**
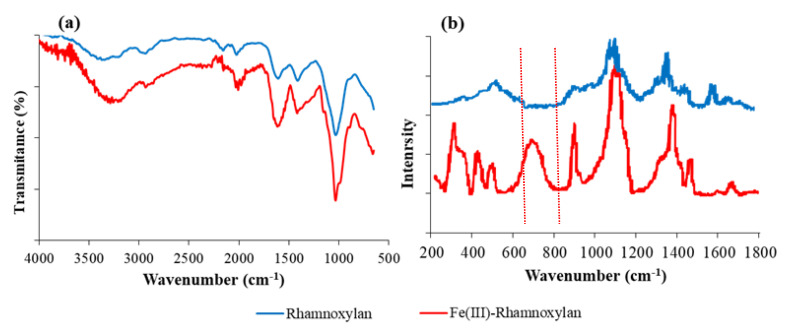
(**a**) FTIR and (**b**) Raman spectra of rhamnoxylan and Fe(III)–rhamnoxylan complex.

The FT-IR spectra of rhamnoxylan and the Fe(III)-rhamnoxylan complex are shown in Figure 1. All the absorption bands of the sugar moieties in rhamnoxylan were present in the spectrum of the iron complex. The characteristic bands due to ν(OH) at 3337–3371 cm^−1^, ν(C–C) in rhamnonosyl side chain at ~1059 cm^−1^ and β-glycosidic C-H bending at ~910 cm^−1^ were observed in both the samples. A broad absorption at 3371 cm^−1^ in the spectrum of the complex appears to be shifted to a higher energy from 3337 cm^−1^ due to ν(O-H) [11] in the pure rhamnoxylan [12,13]. This clearly indicates formation of a coordinate covalent bond between the hydroxyl groups and iron. Slight shift of glycosidic linkage, C-O-C was observed at 1034 cm^−1^ [13,14,15,16]. A Fe-O absorptions below 450 cm^−1^ that falls in the far IR region is generally indicative of formation of new Fe-O bond; this could not be recorded due to instrumental limitation. For that Raman spectrum of the complex was compared with that of the rhamnoxylan shown in Figure 1b and ferric chloride reported in literature [17]. Prominent Raman shifts in the region 300–420 cm^−1^ were observed in the spectrum of the complex that confirmed the existence of Fe-O bond in the complex and the absorption at 255–258 cm^−1^ assigned to an A_1g_(Fe-Cl) mode [17] was absent in our spectrum indicating that the complex was free from FeCl_3_. The AgNO_3_ test also confirmed the absence of Cl¯ ion in the complex.

#### 2.2.4. Structural Analysis of Rhamnoxylan by MALDI-ToF Mass Spectroscopy

**Figure 2:**
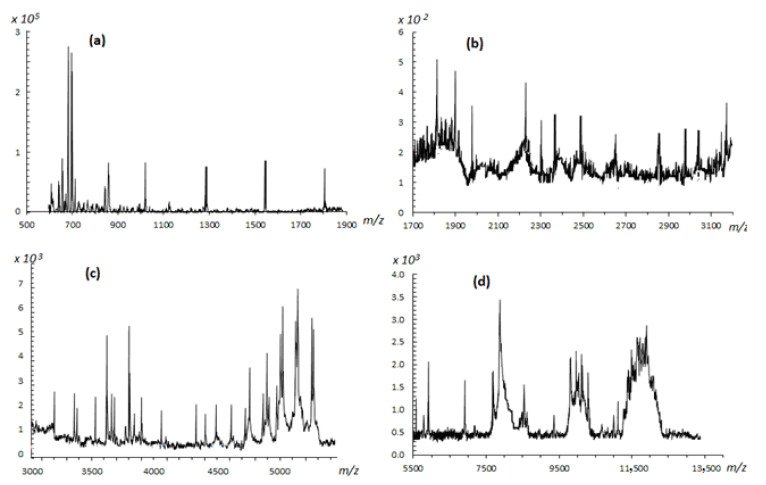
MALDI-TOF mass spectra of rhamnoxylan in (**a**) 500–1900, (**b**) 1700–3100, (**c**) 3000–6000 and (**d**) 5500–14,000 *m*/*z* ranges.

The MALDI-ToF mass spectrum of the water soluble hemicellulose is shown in Figure 2. The assignments of fragment ions were done by mass interval analysis. The masses of residual ions of xylose (M = 150) and rhamnose (M = 164) after glycosidic cleavage (loss of 18 Da) were calculated as (132)_n_ + 23, (132)_n_ + 23 + 18 Da, (146)_n_ + 23 Da, (146)_n_ + 23 + 18 Da and (146)_n_ + (132)_n_ + 23 Da, where n is the number of monosaccharide units and 18 Da the mass of reducing end consisting of two hydrogen and one oxygen atoms. The mass difference between the daughter and the parent ions was used to identify the glycoside position in the monosaccharides. Such fragmentation patterns have been categorized into various types by Domon and Costello [18,19].

High intensity peaks were observed in the 600–900 *m*/*z* range. The intense peak at *m*/*z* 703 was assigned to a [5 Xyl + Na]^+^ adduct, where Xyl is the xylose unit. The neighboring peaks at 643 and 626 *m*/*z* with a mass difference of 60 and 78 Da indicate β-1,4 linkages of xylosyl residues in the xylan backbone due to loss of C_2_H_4_O_2_ and C_2_H_4_O_2_ + 18 (cross-ring A/X-type cleavage). A peak at *m*/*z*~850 represents a [Xyl-Xyl-Xyl-Xyl-Xyl-Rh + Na]^+^ residue, where Rh is the rhamnose unit. Two low intensity signals at 834 and 730 *m*/*z* having mass differences of 16 and 120 Da, respectively, suggest that rhamnose is also linked to xylose through β-1,4-linkage. Relatively higher intensities of these signals indicate that these groups are present as branches at the xylan chain. This is because bulky groups are more vulnerable to fragmentation. A low intensity peak at *m*/*z* 661 was assigned to [Xyl-Xyl-Xyl-Xyl-Xyl + H]^+^ xylosyl residues. The lower intensity signal of the fragment is understandable because the carbohydrates are not easily protonated [20]. Very low intensity peaks were observed in the 900–3500 *m*/*z* range. There is a series of weak signals spaced by 132 Da; around *m*/*z* 1028, 1157, 1290, 1554 (dimer) and 1818.237 (dimer). Above *m*/*z* 1800, the rhamnosyl residues produced peaks frequently.

Some lower intensity peaks at *m*/*z* 1906, *m*/*z* 1894 and *m*/*z* 1866, having mass difference of 78, 90 and 118 Da from *m*/*z* 1984, due to cross-ring fragmentation (A/X-type) suggested that xylosyl and rhamnosyl residues are also linked through β-1,3 positions along with β-1,4. The lower intensities may be indicative of a rigid main chain difficult to undergo fragmentation. In the *m*/*z* 8000–13,000 range bunches of peaks were observed. These bunches most probably consist of a series of signals separated by 132 and 146 Da corresponding to xylosyl and rhamnosyl residues, respectively. Several signals with a difference of 16 and 17 Da were observed due to loss of O and OH, which are abundantly present in the polysaccharide structure. There was no evidence of the presence of uronic acids because of the absence of peaks having a mass difference of 178 Da.

**Figure 3:**
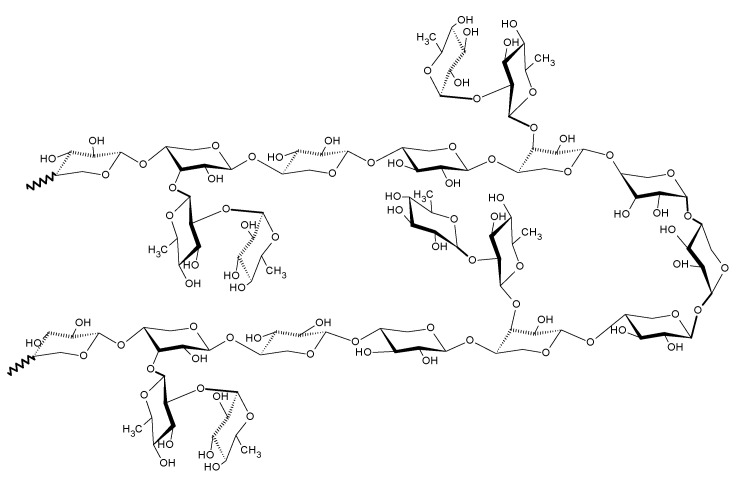
Structure of rhamnoxylan based on MALDI-ToF mass spectral analysis.

This analysis shows that the main chain of polysaccharide consists of β-1, 4-d-xylose linkages branched with β-1, 3-linked rhamnose units. Thus, a block of the polymeric structure of rhamnoxylan can be proposed as shown in Figure 3. According to this, a mole of rhamnoxylan (2.47 × 10^6^ Da) would consist of around 850 such blocks and 61,000 monosaccharide units.

#### 2.2.5. Electronic Absorption Spectrophotometry

**Figure 4:**
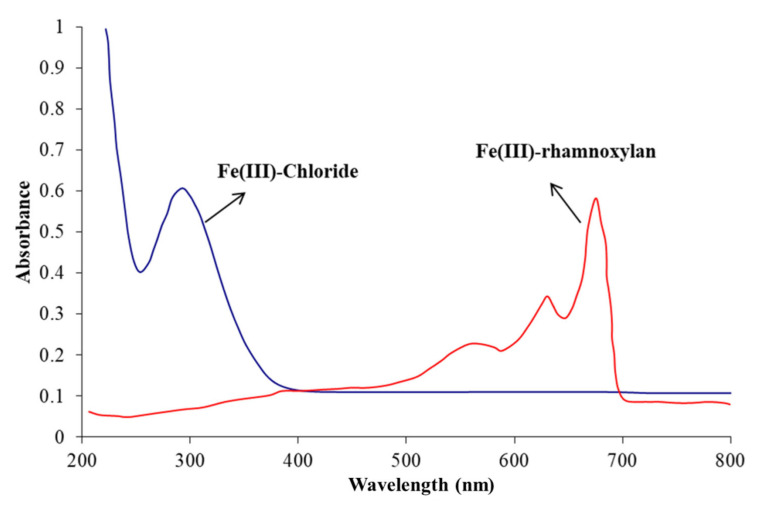
UV-Vis spectra of FeCl_3_·6H_2_O (in water) and Fe(III)-rhamnoxylan (diffuse reflectance).

Electronic absorption spectra of iron complexes in the UV-Visible range provide useful information about the oxidation state of the metal ion, chromophores in the ligand, charge transfer transitions and geometry of the complex ion. High-spin Fe^3+^ complexes contain five unpaired electrons in the d orbital, so all d-d transitions are spin forbidden, hence, should be weak. Most of the Fe^3+^ complexes exhibit weak d-d absorption bands, while some have been reported to show quite intense absorptions. The spectrum of the Fe(III)-rhamnoxylan was characteristic of a complex formation and entirely different from that of FeCl_3_·6H_2_O shown in Figure 4. The spectrum of FeCl_3_·6H_2_O consisted of a single absorption at about 300 nm [21] whereas, that of the complex exhibited three absorptions in the visible region centered at 400 (very broad), 570 (broad), 645 and 690 nm (sharp). This pattern resembles those of six- coordinate Fe^3+^ complexes.

The absence of any absorption in the regions 420–450 nm and 800–900 nm rules out the presence of Fe^2+^ ion in the complex [22]. The assignment of the observed absorptions is straightforward as for the octahedral complexes [23,24,25,26] and can be assigned as: ~400–^6^A_1g_ → (^4^A_1g_, ^4^E_g_); ~570 nm–^6^A_1g_ → ^4^T_2g_; ~645 nm (charge transfer, O-Fe LMCT); ~690 nm–^6^A_1g_ → ^4^T_1g_. The sharpness of low-energy bands in the spectrum of the complex may be attributed to combination vibrational bands of the ligand.

#### 2.2.6. Mössbauer Specroscopy 

**Figure 5:**
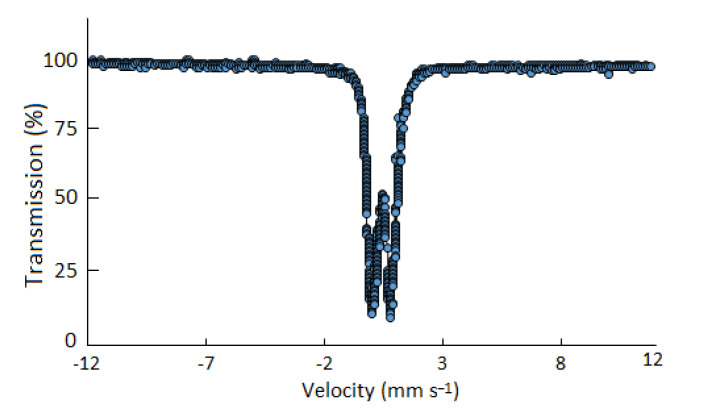
Mössbauer spectrum of Fe(III)–rhamnoxylan complex at room temperature (25 °C).

The Mössbauer spectrum recorded at room temperature is shown in Figure 5. The spectrum was fitted to a single doublet to compare it with a reported iron polysaccharide complex [27]. The ferrous doublet was absent that confirmed the absence of Fe^2+^ ion in the sample. The isomeric shift and quadrupole splitting were found to be 0.35 and 0.71 mm s^−1^, respectively. These values correspond to ferrihydrite iron core [28].

**Figure 6:**
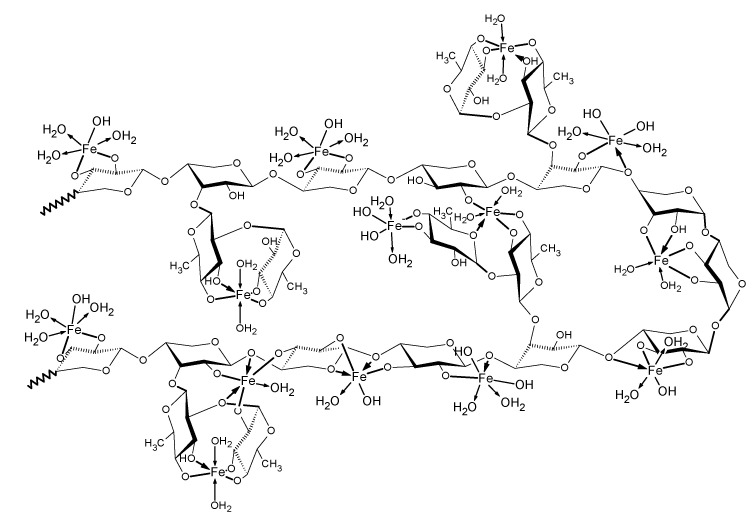
Proposed structure of the Fe(III)–rhamnoxylan complex.

Based on the analytical (elemental and monosaccharide analyses) and spectroscopic (FT-IR/Raman and electronic absorption) evidence presented so far, the coordination sphere of Fe^3+^ ion in the complex appears to have an octahedral geometry, where the macromolecule acts as an anionic ligand. A structural block of the Fe(III)-rhamnoxylan complex can be shown as in Figure 6. Accordingly, a mole of the complex (2.52 × 10^6^ Da) would consist of around 578 such blocks. This indicates that the degree of polymerization has been reduced to ~68% on complex formation. The salient features of the proposed structure are: (i) the rhamnoxylan acts as an anionic ligand under highly alkaline condition, (ii) coordinated water (1–3 molecules per iron atom) is present, (iii) varying number (1–2) of free OH¯ ions in the alkaline medium are also found in the coordination sphere. The structure represents a polynuclear ferrihydrite iron core similar to the *Enteromorpha* polysaccharide-iron (III) complex [29]. The Fe(III)-rhamnoxylan complex pre-dominantly appeared to be hydrophobic (lipophilic) as shown by the solubility data.

#### 2.2.7. Powder X-ray Diffraction Analysis

**Figure 7:**
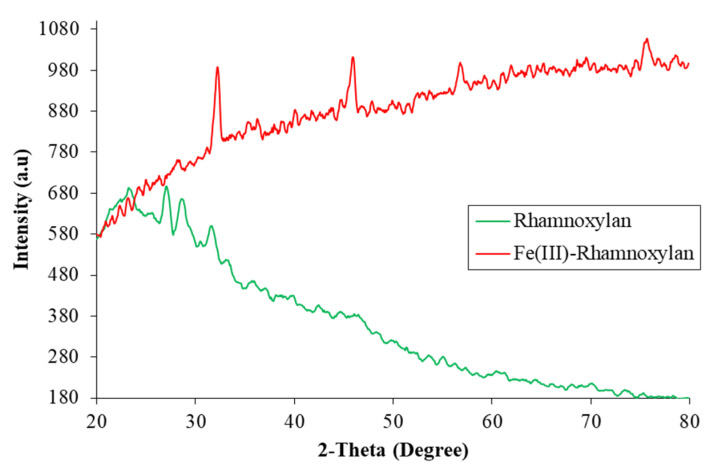
X-ray diffraction patterns of rhamnoxylan and Fe(III)-rhamnoxylan complex.

The p-XRD spectra of rhamnoxylan and the Fe(III)-rhamnoxylan complex are shown in Figure 7. Rhamnoxylan appeared to be mainly in amorphous state, whereas the complex exhibited four peaks at 2θ values 33°, 45°, 57° and 75° indicating a crystalline nature of the complex [30,31,32,33,34] and favoring octahedral geometry. The sharp peak around 33° corresponds to that of iron oxide nanoparticle [31,32,35], whereas at 45° is related to the iron crystal [32]. The appearance of peaks represents different crystal sizes ranging from nano- to micro-meter [23]. The significant difference in the spectra suggests a phase change due to complex formation. These peaks represent different crystal sizes ranging from nano- to micro-meter scale. Attempts to grow a single crystal for further structure elucidation were not successful.

#### 2.2.8. Magnetic Properties

The magnetic moment was found to be 5.9 B.M. that corresponds to the spin only value of 5.92 B.M for Fe(III) complexes. This result confirms the high spin configuration of Fe(III) ion in the complex. Thus, the complex exhibits paramagnetism [33,35,36]; the property that may be exploited in biomedical applications including cell separation, immunoassay, magnetic resonance imaging and drug delivery.

#### 2.2.9. Scanning Electron Microscopy

**Figure 8:**
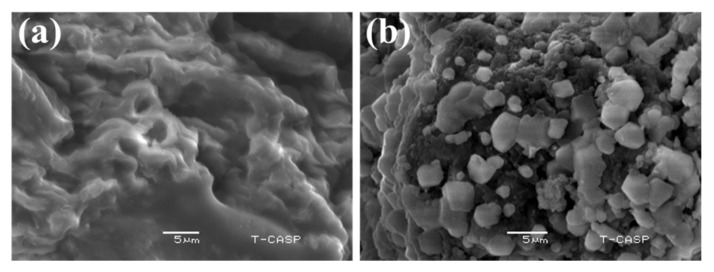
SEM images of (**a**) rhamnoxylan and (**b**) Fe(III)-rhamnoxylan complex.

The SEM images of rhamnoxylan and the Fe(III)-rhamnoxylan complex are shown in Figure 8. A comparison of the two micrographs clearly shows the formation of a new material with change in the morphology after complexation.

#### 2.2.10. Thermal Analysis

**Figure 9:**
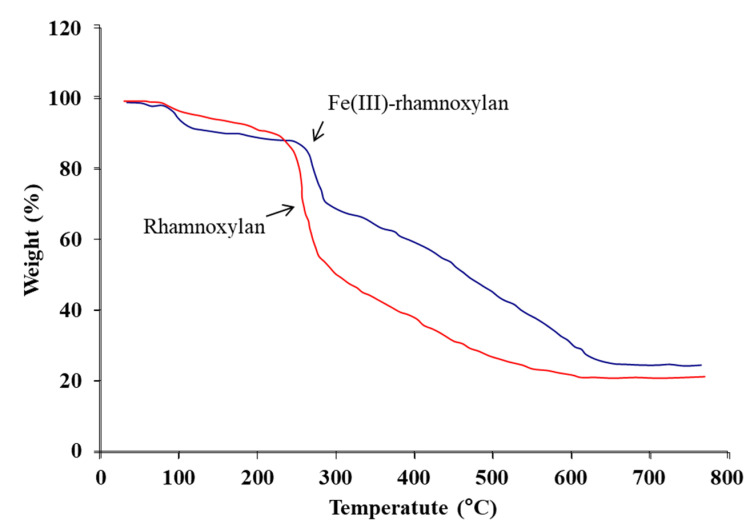
TGA curves of rhamnoxylan and Fe(III)-rhamnoxylan recorded at 10 °C min^−1^ under nitrogen flow (30 mL min^−1^).

TGA was used to determine thermal stability of the complex and to further verify the presence of organic component in it. Thermograms of rhamnoxylan and the Fe(III)-rhamnoxylan are shown in Figure 9. A three-stage decomposition of the complex was observed. The stage I at ambient to ~120 °C corresponds to a loss of absorbed moisture (~11.3%) in the complex; whereas in case of rhamnoxylan this loss was ~5.8% due to absorbed moisture at ~100 °C. It was noted that decomposition of the complex starts at a higher temperature (~271 °C) compared to that of the rhamnoxylan (~225 °C); this suggests that the complex is thermally more stable than the rhamnoxylan. Two major losses occurred at stages II and III, which account for the decomposition of the polymeric ligand. A relatively lower weight loss (~20%) at stage II probably represents side chain decomposition compared with a major loss (~40%) at stage III due to decomposition of the main polymer chain. The residue of the complex was ~26% that corresponds to Fe_2_O_3_.

#### 2.2.11. Electrical Properties

**Figure 10:**
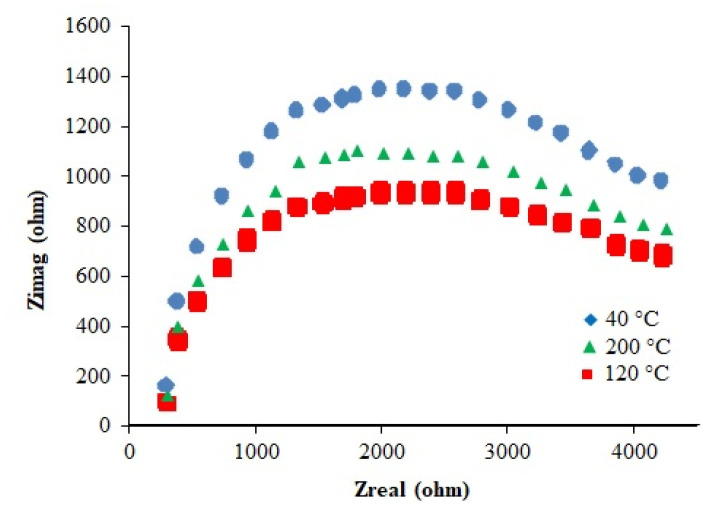
Impedance spectra of the iron complex recorded at 40, 120 and 200 °C.

The impedance spectra of the samples are shown in Figure 10. The resistance and ionic conductivity data at different temperatures are presented in Table 2. It can be seen that resistance decreases and conductivity increases with a rise in temperature in accordance with the kinetic theory. These data show that the complex can be used as a solid polymer electrolyte.

### 2.3. Stability Study

**Figure 11:**
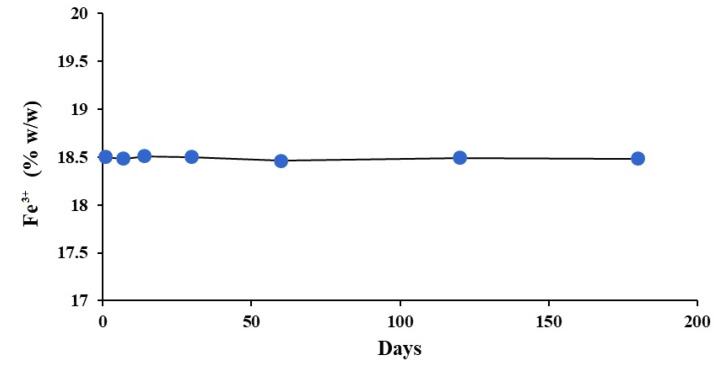
Fe^3+^ content in the Fe(III)-rhamnoxylan complex over a period of six months stored at 50 ± 1 °C and 75% relative humidity.

The results of the six-month accelerated stability study are depicted in Figure 11. It was observed that the Fe^3+^ content remained unchanged throughout the study period and there was no Fe^2+^ impurity detectable in the product. This is understandable because Fe^2+^, the only expected impurity, may be produced under reducing environment and cannot creep up in the presence of air and humidity. Moreover, no significant change in the FT-IR and electronic absorption spectra was observed during this period. So, based on this study and TGA data, it can be concluded that the product is highly stable.

### 2.4. Biological Evaluation

#### 2.4.1. Acute Oral Toxicity

According to OECD guidelines, 24 h after administration of the test dose (5000 mg kg^−1^ body weight) none of the animals was found to be dead and the animals showed no significant change in eye color, hair fall and weight for next 48 h. During that time, they remained active with normal heart and breathing rate and no irritation. The eye reflexes were also positive as assessed by a standard method [37]. No redness or ulceration was found in the stomach walls, indicating a good tolerability needed for long term treatment. The isolated stomach, spleen, liver, intestine, heart and kidneys were found to be normal as examined microscopically. The results of this test suggested that the synthesized Fe(III)-rhamnoxylan complex is non-toxic and may be categorized as ‘unclassified’ according to GHS categorization [38].

#### 2.4.2. In Vivo Absorption and Excretion of Iron

**Figure 12:**
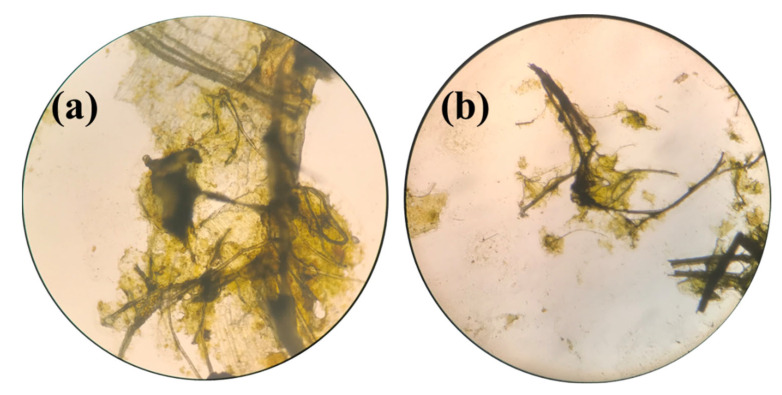
Microscopic image of (**a**) Fe(III)-rhamnoxylan in stomach after 24 h and (**b**) stomach material after 14 days.

After oral administration of 5000 mg kg^−1^ body weight of Fe(III)-rhamnoxylan in the acute toxicity test, the stomach material was microscopically found to contain folds of the hemicellulose (Figure 12a) indicating that the complex remains intact in the stomach and reaches intestine slowly releasing iron there, after dissolution of rhamnoxylan at the alkaline pH in the duodenum. The complex is predominantly lipophilic and slowly dissolves in alkaline medium. Some amount of iron in feces and significant amount in blood were detected indicating that iron from the complex is released and distributed throughout the body. Stomach was almost clear of the complex after 4 days (Figure 12b).

As the storage of iron in the body depends upon several factors including that in stomach, spleen, liver, macrophages, absorption from GIT and simultaneous release through bile [39], the bioavailability of iron cannot be accurately determined from the AUC. So, the bioavailability was assessed as the % increase of iron in blood after 14 days (Table 3) and it was estimated to be ~13.29% that was slightly higher than that of the IPS standard (~9.78%).

#### 2.4.3. Therapeutic Evaluation

**Figure 13:**
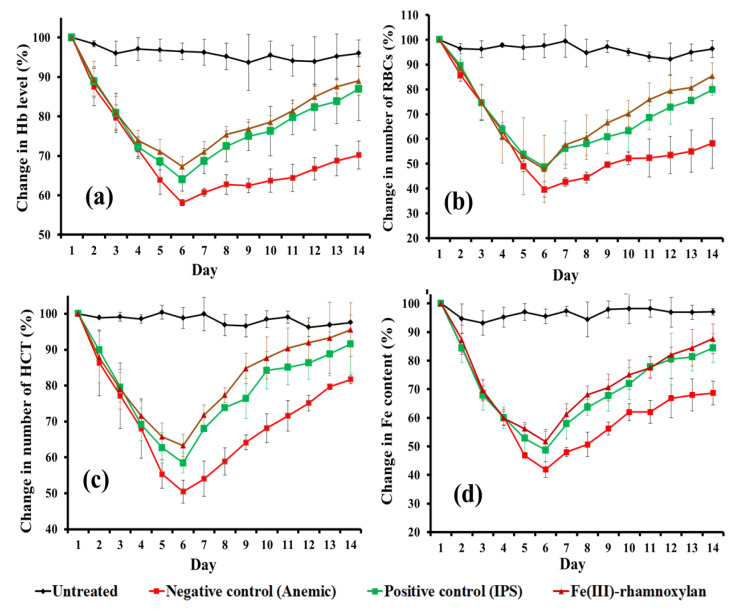
Effect of Fe(III)-rhamnoxylan on (**a**) % Hb, (**b**) % RBCs, (**c**) % HCT and (**d**) Fe content.

The Figure 13a shows the % change in Hb level for 14 days experiment. The minimum levels of Fe, Hb, HCT and RBCs count were observed on the 6th day. However, the minimum levels of all these parameters were better in the treatment groups compared to those for the untreated control (Table 3). A comparison of the Hb levels on the day 14 indicates that recoveries were: 89% (Fe(III)-rhamnoxylan), 87% (IPS) and 70% (negative control/anemic), suggesting that the Fe(III)-rhamnoxylan complex is equally or slightly more effective in raising the Hb level than IPS (the standard drug). Similar trends were observed in RBCs (Figure 13b), HCT (Figure 13c) and blood iron (Figure 13d). Overall, the in vivo study demonstrates better (*p* < 0.05) efficacy, bioavailability and toxicity profile of Fe(III)-rhamnoxylan, in the animal model under investigation, compared with a commercially available standard (IPS). Fe(III)-rhamnoxylan can be considered as a potential candidate for clinical trials.

## 3. Materials and Methods

### 3.1. Materials

Rhamnoxylan from *S. plebeia* (Mw 2.47 × 10^6^ Daltons containing 99.32% Xylp and 0.68% Rhap) was used from a sample previously prepared and characterized in our laboratory [40]. Ferric chloride hexahydrate (FeCl_3_·6H_2_O), silver nitrate (AgNO_3_) sodium hydroxide (NaOH), hydrochloric acid (HCl, 37%) and 2,5-dihydroxybenzoic acid (DHB), all were from Sigma-Aldrich, St. Louis, MO, USA. IPS (Italfarmaco S.p.A. Milan, Italy) was used as the standard drug and manganese chloride (MnCl_2_, QQ 153, Sherwood Scientific Ltd., Cambridge, UK) was used as the calibrating standard for magnetic measurements. All the chemicals were of analytical grade and used without further purification. Distilled water was used throughout the experimental work.

### 3.2. Synthesis of Fe(III)-Rhamnoxylan Complex

Rhamnoxylan (2 g) was suspended in hot water (at 75–80 °C) and allowed to soak for about an hour under constant stirring by a magnetic stirrer. To the stirred suspension, FeCl_3_·6H_2_O (5.35 mL of 75% aqueous solution) was added followed by dropwise addition of NaOH (40% aqueous solution) to adjust the pH to 11.5. The mixture was stirred for further 60 min at 80 °C. The brown colored complex formed as precipitate was isolated by filtration under vacuum. The complex was washed with dilute HCl to remove any ferric hydroxide that may have formed followed by several washings with methanol. The complex was finally washed with ether and dried at 60 °C in an oven and ground into a powder (yield 1.8 g; 90% of the rhamnoxylan used). The complex formation occurred in the pH range 8–11.5 with better yield at 11.5. Similar pH has been used in the synthesis of an iron-saccharide complex [41]. The purity of the complex was ascertained by the single peak appearing in the gel permeation chromatogram.

### 3.3. Characterization 

The elemental (carbon and hydrogen) analysis of the synthesized complex was carried by Leco 4201 elemental analyzer (Leco, St Joseph, MI, USA). Iron was determined by atomic absorption spectrophotometery. For this, the complex (100 mg) was digested in aqua regia (15 mL) followed by dissolution of the iron salt in water to make 100 mL of the solution. The solution was filtered and analyzed by flame atomic absorption spectrometer AAS FS240 (Varian, Palo Alto, CA, USA) using iron hollow cathode lamp set at 248.3 nm and an air-acetylene flame. The standards (prepared from 1.0 mg mL^−1^ stock solution, lot# D2-FE03121 in 2% HNO_3_ *v/v*, from Inorganic Ventures, Christiansburg, VA, USA Cat# AAFE1-5) and samples were analyzed in triplicate. The R^2^ value of the six-point calibration curve was 0.9998. The AgNO_3_ test was performed to detect Cl^−^ ions if any. Moisture content of the complex was determined by Karl-Fischer titration using Titrino 701KF (Metrohm, Herisau, Switzerland) auto titrator.

Monosaccharide analysis was carried out after hydrolysis of the rhamnoxylan and the iron complex with sulfuric acid and electro chemical detector according to a reported method [42]. Rhamnose and xylose (Sigma Aldrich, St. Louis, MO, USA) were used as standards. The chromatographic conditions were: isocratic elution using 95% water and 5% 0.2 M NaOH at room temperature (25 ± 1 °C); flow rate 0.5 mL min^−1^; injection volume 50 µL.

Molar masses were determined by gel permeation chromatography (GPC) using Agilent 1200 series (Agilent, Waldbronn, Germany) system equipped with PL aquagel-OH mixed column and refractive index detector (G1362A) using DMSO containing 0.50% LiBr as eluent (flow rate 0.5 mL min^−1^ at 70 °C) and injection volume of 10 μL. Pullulan (96351 Supelco standard set Mp ~350–700,000) and dextran (31430 Supelco standard set Mp 1000–400,000) were used as calibration standards.

The FT-IR spectra were recorded at 2 cm^−1^ resolution, in transmission mode, using a Carry 630 FTIR spectrophotometer (Agilent Technologies, Santa Clara, CA, USA). The powdered sample was placed on the diamond crystal and the spectrum was recorded in the 4000–630 cm^−1^ range. Raman spectra were recorded by use of a reflex microscope (inVia™ confocal Raman microscope, Renishaw). A near-infrared diode laser was used for excitation at 785 nm. Electronic absorption spectra were recorded in diffuse reflectance mode by UV-1800 spectrophotometer (Shimazdu, Kyoto, Japan) in the 200–800 nm range.

MALDI-ToF mass spectrometric analysis was carried out to determine structural features of the rhamnoxylan used for complex formation. This technique is appropriate as it produces singly charged ions; this has been demonstrated in our recent work [20]. The spectra were recorded in the 500–14,000 *m*/*z* range by MALDI-ToF/ToF (Bruker Daltonics Inc, Fremont, CA, USA) spectrometer using single ToF option in the reflectron mode. The desalted sample (1.5 mL) was spotted in triplicate on MTP 384 polished steel BC targets (Bruker Daltonics) and the samples were overlaid with 2,5-DHB (1.5 mL) and dried. The rest of the procedure was same as reported earlier [20].

The Mössbauer spectrum was recorded at room temperature using a 25 mCi ^57^Co(Rh) source. The velocity was calibrated by laser interferometry. The powdered sample (~125 mg) was pressed in a brass ring with area of 1.75 cm^2^ and sealed with benzene-styrofoam. Aluminum foil was used as the backing. The spectrum was fitted to single doublet assuming a Lorentzian line shape.

Powder X-ray diffraction (p-XRD) spectra were recorded by D2 PHASER diffractrometer (Bruker, Bremen, Germany). The samples were tightly packed in the sample holder and diffractions were recorded using Cu radiation (1.54184 Å, 30 kV, 10 mA) and Lynxeye array detector over the 19°–80° 2θ range.

Magnetic susceptibility measurements were performed by using MSB MK 1 magnetic susceptibility balance (Sherwood Scientific, Cambridge, UK). The balance was calibrated by using MnCl_2_, standard (QQ153, Sherwood Scientific Ltd., UK). The effective magnetic moment (µ_eff_) was calculated by the formula: µ_eff_ (B.M.) = 2.828(χAT)^1/2^, where χA is the atomic susceptibility of the paramagnetic ion (Fe^3+^ in this case) corrected for the ligands and T is the absolute temperature.

Surface morphology was studied by scanning electron microscopy (SEM) using JEOL JSM-6480LV microscope after sputter coating with gold (in case of rhamnoxylan) and without sputter coating (in case of Fe(III)-rhamnoxylan as the complex was self-conducting due to the presence of iron in it). For this, samples were fixed on the stub with a double-sided adhesive tape and images were recorded at different magnifications.

Thermal analysis was carried out in TGA mode by SDT Q600 analyzer (TA Company, La Vergne, TN, USA). Samples (approx. 6 mg) were heated in aluminum sample pans from 10 °C to 700 °C at a heating rate of 10 °C min^−1^ under nitrogen flow (30 mL min^−1^).

To determine electrical properties, sample was cold-pressed into cylindrical pellets (10 mm dia) at 40 MPa. The pellets were painted with silver paste and impedance spectra were recorded in air at 40–200 °C (heating rate: 15 °C) using Gammry Interface 1000 potentiostat. The total resistance was determined by the formula: *R_t_ = R_g_ + R_gb_*, where *R_g_* and *R_gb_* are the resistance of grain interior and grain boundary, respectively. The ionic conductivity (σ) was calculated using the formula: σ = *l/RA*, where *l* is the thickness of the sample and *A* the cross-section area.

A six-month accelerated stability study was performed, according to WHO Q1F Stability Guideline [43] as follows. Three different batches of the complex contained in plastic PP Securitainers^®^ (VWR International Ltd., Lutterworth, UK) were stored at 50 ± 1 °C and 75% relative humidity and assayed for Fe^3+^ according to a validated method [44]. In addition to this the FT-IR and electronic absorption spectra were also recorded before and after six months.

### 3.4. Biological Evaluation

The synthesized complex was subjected to acute toxicity test and therapeutic evaluation in vivo in standard Dutch rabbit. These studies were approved by the institutional review board (vide IRB-124/12-2018) of the Forman Christian College (A chartered University), Lahore, Pakistan. All animal experiments complied with the ARRIVE guidelines in accordance with the U.K. Animals (Scientific Procedures) Act, 1986 and associated guidelines (EU Directive 2010/63/EU for animal experiments).

#### 3.4.1. Acute Oral Toxicity Test

This test was performed according to OECD guidelines for determination of oral toxicity [38]. Ten male rabbits (800–1000 g) were fed with fodder and free water adaptively for a week. Five of the animals were orally fed with the synthesized complex at a dose of 5000 mg kg^−1^ body weight, while the other group of five was kept as control. Both the groups were monitored for 48 h for any physical and behavioral changes including eye color, eye reflexes, hair fall, weight change, heart rate, breathing rate, irritability and activity. Eye reflexes were examined by flashing light into the side of the rabbit’s eye. Corneal reflexes were examined by smoothly touching the cornea with a clean cotton swab to check for any induced blink reflex [37].

#### 3.4.2. Therapeutic Evaluation

Twenty male rabbits (6–8 weeks old, weighing 1000 ± 20 g) were purchased from Tollinton Market, Lahore, Pakistan and housed in the animal storage facility at Forman Christian College. They were fed with plain water and a low iron diet, adaptively for seven days. The animals were divided equally into four groups as: A (untreated), B (negative control/anemic), C (positive control/IPS) and D (Fe(III)-rhamnoxylan). Iron-deficiency anemia was induced as reported earlier [45] by oral administration of 11 µg kg^−1^ per day phenylhydrazine for two days. The treatment was started after 4th day of the induction of anemia. The groups C and D were administered, orally, a dose of the standard (IPS) and the test sample equivalent to Fe 33 mg kg^−1^ body weight, respectively [46]. Blood samples (1mL) were collected over 14 days with an interval of 24 h from the marginal ear vein, using a 1 mL disposable syringe (with the needle 26G × 5/8 in.) containing K_2_-EDTA, The effect of phenylhydrazine on Hb, RBCs, HCT and Fe levels was continuously monitored separately. The percent change in the values was plotted against time (days), where % change was calculated by the equation:

$$\%\;change=\left[\frac{100-100\times \left(Initial\;value\;of\;the\;parameter-Value\;on\;the\;particular\;day\right)}{Initial\;value}\right]$$

### 3.5. Statistical Analysis

The student *t* test was applied to observe the significant difference between the negative control (anemic) and the Fe(III)-rhamnoxylan group; the significance was determined at *p* < 0.05.

## 4. Conclusions

A molecular complex of Fe^3+^ with rhamnoxylan from *Salvia plebeia* seeds having the octahedral coordination environment and high magnetic susceptibility was successfully prepared. The complex exhibited good electrolytic properties and accelerated stability. The Fe(III)-rhamnoxylan was non-toxic and exhibited better hematinic property as compared with iron protein succinylate (a commercially available product) with no gastric irritation. This provides for a safer potential candidate as hematinic for clinical trials.

## Figures and Tables

**Table 1:** Elemental, moisture, monosaccharide and GPC analysis data (% *w/w* on dry substance basis).

Compound	Carbon	Hydrogen	Iron	Moisture	Rha*p*	Xyl*p*	M_w_ (Dalton)
Rhamnoxylan	46.73 ± 0.04	6.45 ± 0.02	-	5.98 ± 0.01	0.68 ± 0.02	99.32 ± 0.05	2.47 × 10^6^
Fe(III)-rhamnoxylan	31.1 ± 0.03	5.15 ± 0.01	18.40 ± 0.02	11.0 ± 0.01	0.61 ± 0.01	89.12 ± 0.04	2.52 × 10^6^

**Table 2:** Resistance and conductivity data of the iron complex at different temperatures.

Temperature (°C)	*R_g_* (Ω)	*R_gb_* (Ω)	*R_t_* (Ω)	σ (S cm^−1^)
40	205.35	1650.46	1845.64	2.51 × 10^−4^
120	100.86	456.24	507.21	1.01 × 10^−3^
200	36.32	65.89	107.21	4.99 × 10^−3^

**Table 3:** Bioavailability data of Fe(III)-rhamnoxylan and IPS.

Treatment	Amount of Fe(III) in Control Animals after 14 Days (mg)	Amount of Fe(III) in Blood after 14 Days (mg)	Bioavailability (% Increase)
IPS (33 mg equivalent of iron)	6.4	7.02 ± 0.3	~9.78
Fe(III)-rhamnoxylan (33 mg equivalent of iron)	6.4	7.26 ± 0.2	~13.29
